# Probabilistic Inference with Polymerizing Biochemical Circuits

**DOI:** 10.3390/e24050629

**Published:** 2022-04-29

**Authors:** Yarden Katz, Walter Fontana

**Affiliations:** 1 Digital Studies Institute, University of Michigan, Ann Arbor, MI 48109, USA; 2 Department of Systems Biology, Harvard Medical School, Boston, MA 02115, USA

**Keywords:** probabilistic inference, molecular information-processing, single-celled organisms, biological computation, changing environments

## Abstract

Probabilistic inference—the process of estimating the values of unobserved variables in probabilistic models—has been used to describe various cognitive phenomena related to learning and memory. While the study of biological realizations of inference has focused on animal nervous systems, single-celled organisms also show complex and potentially “predictive” behaviors in changing environments. Yet, it is unclear how the biochemical machinery found in cells might perform inference. Here, we show how inference in a simple Markov model can be approximately realized, in real-time, using polymerizing biochemical circuits. Our approach relies on assembling linear polymers that record the history of environmental changes, where the polymerization process produces molecular complexes that reflect posterior probabilities. We discuss the implications of realizing inference using biochemistry, and the potential of polymerization as a form of biological information-processing.

## 1. Introduction

Probabilistic inference—a procedure for estimating unobserved variables in probabilistic models—has been used to describe various aspects of cognition [1,2]. In this line of work, organisms are thought to build probabilistic models of their world and use these to guide action and perception via inference. Although the work on biological instantiations of probabilistic inference has focused on animal nervous systems [3,4], single-celled organisms also show complex, history-dependent behaviors in changing environments. This history-dependent character has been described in cognitive terms, such as “learning,” “memory,” and “decision-making” [5,6,7,8,9,10,11,12,13,14,15].

The internal state of microbes, for instance, is shaped by past experiences [16,17,18]. *B. subtilis* populations exposed to distinct environmental perturbations and then grown in the same environment can be distinguished (based on gene expression) for as long as 24 h after the perturbation, suggesting that cells retain a “memory” of past environments [16]. *E. coli* cells grown in an environment that switches periodically from glucose to lactose eventually adapt to the switches and maintain a consistent growth rate through the switches [17]. Yeast strains anticipate their environment by producing a metabolic program needed to metabolize an alternative nutrient, even before the preferred nutrient is depleted [19]. All these behaviors occur on an ontogenetic timescale, much faster than that of mutation and natural selection.

These observations raise the question of whether probabilistic inference can be realized in cellular biochemistry. In principle, Chemical Reaction Networks (CRNs), commonly used to formalize biochemical systems, can approximate any computable function [20,21]—and therefore implement inference. Several studies have shown how CRNs can implement probabilistic inference [22,23,24,25,26]. For instance, [22] showed it is possible to construct a CRN that, at steady-state, encodes the joint probability distributions of probabilistic models known as factor graphs, and suggested enzyme-free DNA strand displacement [27] as a physical realization. However, strand displacement is an unlikely cellular mechanism. Moreover, cells act as the environment changes [13,24], and while the abundances of cellular components change in a stochastic manner [28,29], which raises an important question: which biologically plausible, cellular mechanisms can support inference under such stochastic conditions? Some studies have explored phosphorylation and transcriptional regulation as vehicles for forming memories and simple associations. For example, [10] designed biochemical circuits, which use either phosphorylation or transcriptional control, that can be conditioned through a unicellular analog of Hebb’s rule, while [30] evolved chemical circuits *in silico* for associative learning. Such mechanisms could potentially be used to build inference-performing circuits.

Yet biochemistry also includes generative and combinatorial mechanisms, such as polymerization, which are not part of the typical repertoire of molecular mechanisms (e.g., protein phosphorylation or transcriptional regulation) that are thought to “compute” or “process information”. Through polymerization, the cell produces structures such as microtubules and actin cables, which are highly regulated [31] and crucial to a variety of cellular functions. While these polymers are often studied for their mechanical properties [32], it has also been recognized since the early days of molecular biology that polymerization can be viewed as a computational process, capable of implementing logical automata [33] (more recent studies have also explored the computational power and properties of polymers; see [34] and references therein).

In this paper, we follow a similar line of thought, by exploring how the process of polymer assembly can be used to realize probabilistic inference. We show how the process of linear polymer assembly can be used to perform inference in a Markov model. Our circuit uses the polarity of polymers and their constituents to create a molecular record of the environment’s history (its past sequence of changes), which we show can be used to anticipate the environment’s dynamics and regulate other biochemical programs. This suggests that polymerization—due to its ability to produce macromolecules out of many combinations of parts—can be a useful motif of biological computation. The paper is organized as follows. We first introduce the use of probabilistic inference to anticipate an environment that changes according to a Markov model, and derive the representations and operations needed to approximately implement inference in real-time in such an environment. We then describe a circuit that can perform these operations by assembling linear polymers. We describe several properties of the circuit, and show how the probabilistic information it records can be used to regulate a specific response to environmental change. We close with a discussion of the properties of this circuit and potential future directions.

## 2. Results

### 2.1. Real-Time Inference in Markov Chemical Environments

Probabilistic models have been used to represent a variety of dynamic, uncertain environments. One of the simplest probabilistic models is the discrete-time, finite-state Markov model, in which the state of the environment at time *t* is assumed to depend only on the prior states going back to the t−k time point (where *k* is the order of the model; when k=1, we have a first-order Markov model). While this Markov assumption is violated by many natural processes [35], it will serve as a useful idealization for understanding how biochemical circuits that perform inference can be constructed.

**Figure 1:**
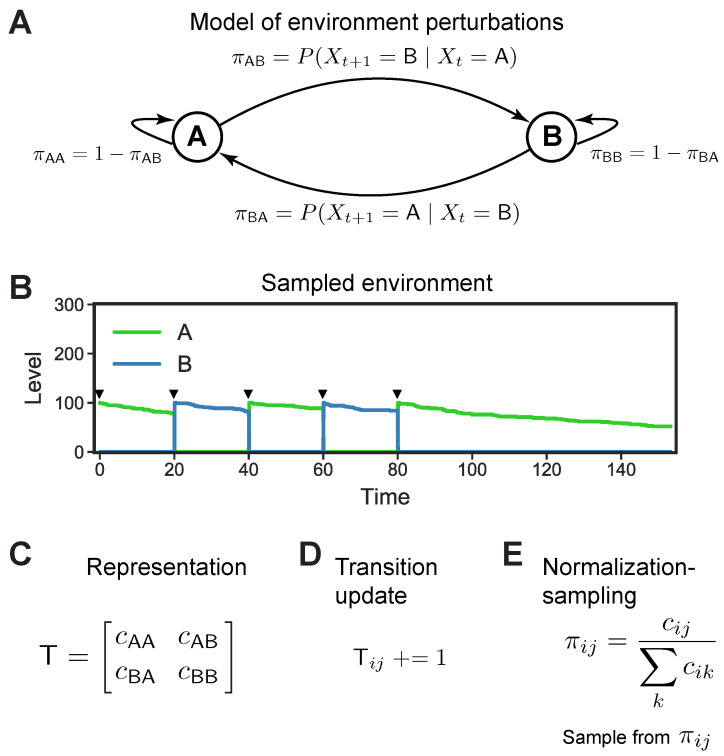
Computational representations and operations for inference in Markov environments. (**A**) A discrete-time Markov model that switches between two states, *A* and *B*, parameterized by two transition probabilities, $\pi_{AB}$ and $\pi_{BA}$. Note that the probabilities of staying in the same state (self-transitions, not shown) are derived from these two parameters ($\pi_{AA}=1-\pi_{AB},\pi_{BB}=1-\pi_{BA}$). (**B**) An environment generated using the model shown in (**A**). At fixed 20 time unit intervals, 100 units of *A* or *B* are pulsed in (after removing any prior *A* or *B*) and allowed to degrade (and/or be consumed by the circuit; hence the diminishing abundances of *A* and *B*). Pulses denoted by arrow heads. (**C**) Transition matrix T that represents the sufficient statistics for the model shown in (**A**). Given the length of the sequence *n* drawn from the model, the sufficient statistics are the number of switches from *A* to *B*, and the number of switches from *B* to *A*. (**D**) Transition operation on T, which increments the appropriate counter when the environment switches from state *i* to *j* (e.g., *A* to *B*). This operation is needed for implementing inference in the model. (**E**) Normalization-sampling operation, which normalizes a row in T (converting counts to probabilities) and samples an entry in proportion to its probability.

We consider a changing environment that can be in one of two states, *A* or *B*, and where switches between states are driven by the Markov model. Such models are parameterized by two *transition probabilities*: the probability of switching from *A* to *B*, $\pi_{AB}$, and the probability of switching from *B* to *A*, $\pi_{BA}$—as shown in Figure 1A. The transition probabilities are unobserved. Through probabilistic inference, one could estimate the values of these probabilities, and use this information to anticipate the environment’s switches—potentially in a way that would be useful to an organism adapting to a changing environment.

To guide our search for biological mechanisms that could instantiate inference in this model, we analyze the task of inference in this model through David Marr’s three levels scheme [36]. Marr’s scheme is a general “top-down” approach to understanding biological computation that begins by describing the *computational level* (level 1): what are the “inputs” and “outputs” to the task, and constitutes a “correct” solution? Given this computational formulation of the task, the *algorithmic level* (level 2) asks for a suitable representation and procedure for carrying out the task. Finally, the *hardware level* (level 3) asks for biologically plausible mechanisms that can realize the representation and algorithm from level 2. The idea is that each level guides and constrains the next level. We will apply this approach to derive the computational representations and operations required for inference in our probabilistic model (levels 1 and 2), and then construct a biological circuit guided by these requirements (level 3).

We first constructed a *Markov chemical environment* whose dynamics are driven by the Markov model shown in Figure 1A. We can think of this chemical environment as a bioreactor in which organisms grow, and where *A* and *B* are analogous to nutrients which are flowed into the reactor by an experimenter and consumed by circuit. The Markov chemical environment is created as follows. We used sequences sampled from the discrete-time Markov model to add/remove *A* and *B* at fixed time intervals, as follows. Initially, a state $X_{0}\in \left\{A,B\right\}$ is sampled from the Markov model and a fixed amount of $X_{0}$ is added to a reactor which contains only our circuit (no *A* or *B*). After a fixed time interval, we sample $X_{t+1}$ from the model given the previous state $X_{t}$. If $X_{t+1}=X_{t}$, no perturbation is performed; otherwise, we remove all *A* and *B* present in the reactor and add $X_{t+1}$. An environment generated by this procedure using a Markov model where $\pi_{AB}=\pi_{BA}=0.95$ is shown in Figure 1B.

Assuming the environment’s perturbations are generated by a Markov model, probabilistic inference can be used to anticipate the environment’s states. In particular, it is useful to compute the probability of encountering *A* or *B* next given past observations of the environment’s states, $\begin{array}{c} \mathrm{history}= \end{array}\langle X_{t},X_{t-1},X_{t-2},\ldots \rangle$, where $X_{t}$ corresponds to the state of the environment at time *t*. In Bayesian terms, anticipation of the environment means computing the posterior predictive distribution, $P(X_{t+1}|\begin{array}{c} \mathrm{history}) \end{array}$. This computation is complicated by the fact that the transition probabilities $\pi_{AB}$ and $\pi_{BA}$ are unknown. One option in such cases is to place prior probabilities on these parameters and then integrate them out to calculate, $\int P\left(X_{t+1}|\begin{array}{c} \mathrm{history}, \end{array}\pi_{AB},\pi_{BA}\right)d\pi_{AB}d\pi_{BA}$. We take an alternative and sometimes simpler strategy of estimating the unobserved transition probabilities, $\pi_{AB}$ and $\pi_{BA}$, through Bayesian inference, and use these estimates to anticipate the next state:

(1) $$P(\pi_{AB},\pi_{BA}|\begin{array}{c} \mathrm{history}) \end{array}\propto P(\pi_{AB},\pi_{BA}|\begin{array}{c} \mathrm{history}) \end{array}P\left(\pi_{AB},\pi_{BA}\right)$$

If a mathematically convenient prior distribution is chosen for $P(\pi_{AB},\pi_{BA})$, then Equation (1) can be solved exactly (see Appendix A). Using the posterior distribution, we can then obtain estimates of the transition probabilities, ${\hat{\pi }}_{AB},{\hat{\pi }}_{BA}$, and use these to anticipate the next state by sampling $X_{t+1}∼P\left(X_{t+1}|X_{t},{\hat{\pi }}_{AB},{\hat{\pi }}_{BA}\right)$:

$$P\left(X_{t+1}=A|X_{t},{\hat{\pi }}_{AB},{\hat{\pi }}_{BA}\right)=\left\{\begin{array}{cc} 1-{\hat{\pi }}_{AB} & \mathrm{if}X_{t}=A\\ {\hat{\pi }}_{BA} & \mathrm{if}X_{t}=B \end{array}\right.$$

An important complication in our setting is that cells act while the environment is changing, so the posterior distribution (Equation (1)) must be estimated in real-time. However, this computation simplifies considerably when we consider the representation and algorithm needed to estimate the posterior distribution (Marr level 2). Crucially, the sequence of observations about the environment, history, does not need to be stored in full; it can be compressed into a matrix T of transition counts (Figure 1C). Assuming *n* possible states of the environment, T is an *n* × *n* matrix whose ith row indicates the number of times the environment switched from the ith state to each of the states. Since the sum of the *i*th row must equal the total number of transitions from state *i* that have been observed, the matrix can be summarized by *n*^2^ − *n* entries. In an environment with two states, the sufficient statistics are simply two counts: the number of times the environment switched from *A* to *B*, and from *B* to *A* (along with the total number of transitions; see Appendix A for details).

Using these counts as the representation, the following elegant algorithm for computing the posterior distribution in real-time emerges. As the environment changes, update the relevant counts in T. Then anticipate the next state given the current state *i* by: (1) normalizing the *i*th row of T to convert counts into probabilities, and (2) sampling a state from that row. This algorithm relies on a representation of counts (as stored in T), and two computational operations. First, it is necessary to update the counts by a *transition update* operation (Figure 1D) that distinguishes *A* to *B* transitions from *B* to *A* transitions. Second, in order to use these counts in downstream computations, we need to be able to convert the counts into probabilities by normalization and sample from the resulting distribution; this is the *normalization-sampling* operation shown in Figure 1E. With these three ingredients—the transition counts from T, an operation that updates the transition counts, and an operation that normalizes and samples from a row of T—the posterior distribution can be estimated in real-time.

### 2.2. Using Polymer Assembly to Represent a Changing Environment

To realize inference biochemically based on the above analysis, we need a biochemical circuit that can perform the operations shown in Figure 1C–E. Such a circuit would need a representation of the transition matrix T, which encodes the number of switches between relevant states of the environment. Two operations on T would then need to be realized molecularly: (1) the ability to count directionally, i.e., to record when the environment has switched from state *A* to *B* (as opposed to from *B* to *A*) and store this information in T, and (2) the ability to access a row of counts in T, normalize it, and sample from the resulting probability distribution. This is challenging since directional counting requires recording a potentially unbounded number of switches in the environment. Some digital counters proposed in the synthetic biology literature, such as [37], have a fixed capacity—determined by the number of genetically components—and are thus not suitable.

We instead constructed a biological realization of the counts matrix and its operations using polymerization. We make use of the idea that the process of assembling a macromolecule such as a polymer can be seen as a computational process. The rules that say how to add a new part (monomer), $X_{n+1}$, to an existing polymer *X*_1_-*X*_2_⋯*X_n_*, depending on the state of the new part $X_{n+1}$ and the configuration of the existing polymer’s end, $X_{n}$, describe a logical flow [33]. A simple example would be the rule: if $X_{n+1}\neq X_{n}$, where $X_{n+1}$ is a monomer, then add $X_{n+1}$ after $X_{n}$. If two types of monomers are available, e.g., $X_{i}=A$ or $X_{i}=B$, then this process would generate alternating polymers such as *A*-*B*-*A* or *B*-*A*-*B*-*A*. The polymerization process, then, is akin to running a kind of logical automata, and the resulting polymer’s sequence is a record of the computation, similar to the output tape of a Turing machine. In our case, however, the assembly process is not sequential and deterministic (as it is in a Turing machine), but rather driven by concurrent, stochastic biochemical reactions.

We use polymerization to represent directional changes in the environment by building a set of linear polymers, which we call *transition history polymers*, made up of *A* and *B* molecules. Each polymer represents the sequence of transitions that have occurred in the environment. The process of assembling the polymer will allow us to count the number of environmental transitions.

**Figure 2:**
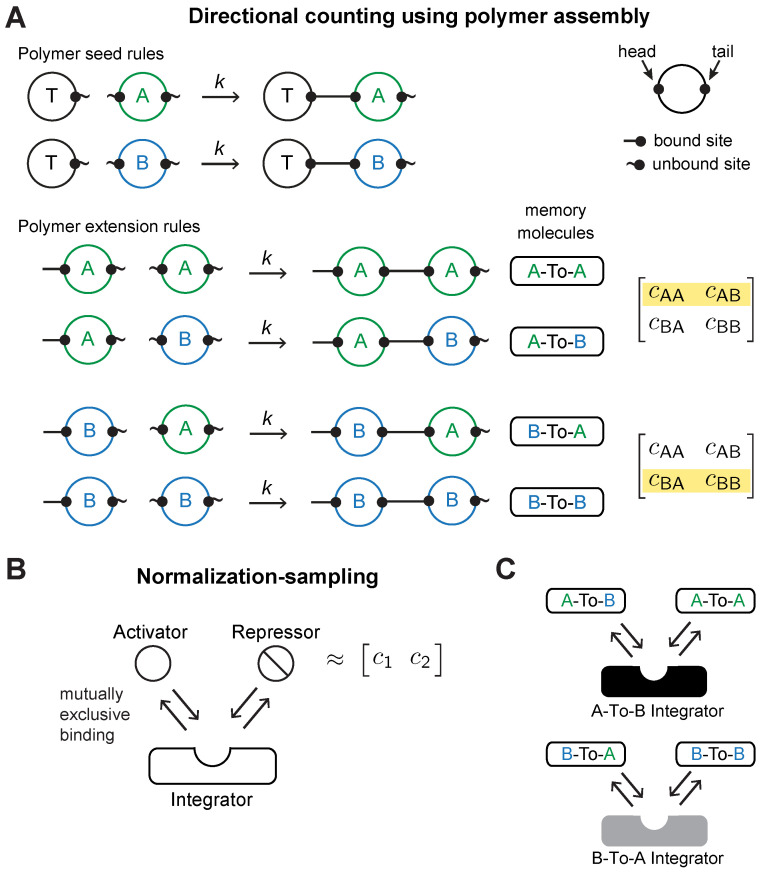
A polymerizing circuit for inference in a Markov model. (**A**) Rules for assembling transition history polymers. Solid line attached to a binding site, indicates a bond, squiggle indicates an unbound site. Top: rules that seed the polymer by creating dimers between a *T* monomer and an *A* or *B* monomer. Bottom: Rules that extend the polymer and record directional changes by producing the appropriate memory molecule ex nihilo. On right, the row of the Markov model’s transition matrix handled by each pair of polymerization rules. All polymerization reactions proceed irreversibly with rate *k*. (**B**) Normalization-sampling operation for converting a row of counts ($c_{1}$ and $c_{2}$) from a transition matrix into a probability vector and sampling an element from it. An Activator and Repressor protein bind a site on an Integrator, in mutually exclusive fashion. (**C**) Normalization-sampling circuit for the A-To-B and B-To-A integrators. Transition-encoding memory molecules act as “activators” and duration-encoding memory molecules as “repressors”.

The transition history polymers are assembled as follows. We assume that the *A* and *B* molecules have a distinguishable “head” site and “tail” site, and that each polymer is built starting from a *T* monomer (which also has a head and tail site). Polymerization then proceeds according to two sets of rules: (i) an unbound *T* can bind the head of a free *A* or *B* molecule (i.e., those molecules whose head and tail sites are unbound), forming a TA or TB dimer (Figure 2A, top); and (ii) the unbound tail of a TA or TB dimer can bind the head of a free *A* or *B* (Figure 2A, bottom). This second set of rules records the direction of change by producing *memory molecules* corresponding to each of the four possible transitions: A-To-A memory molecule upon *A* to *A* binding, A-To-B upon *A* to *B* binding, B-To-A upon *B* to *A* binding, and B-To-B upon *B* to *B* binding. For simplicity, we assume all these polymerization rules are irreversible and proceed according to the polymerization rate constant *k*. The process of polymer assembly thus implicitly “senses” the environment, and uses the polarity of the polymer’s constituents to record the direction of change. (Note that if *A* and *B* are viewed as nutrients, e.g., two different kinds of sugars consumed by microbial cells a bioreactor, we cannot simply assume that these molecules could polymerize into arbitrary linear polymers. However, we could always posit a third “scaffold” protein, which binds *A* and *B*, and can differentially polymerize based on its binding state, using the rules in Figure 2A. For simplicity, we assume *A* and *B* have those polymerizing capacities directly).

As a consequence of polymerization, the abundance of A-To-B and B-To-A will encode the number of transitions from *A* to *B* and *B* to *A*, respectively, while the abundance of A-To-A and B-To-B will correspond to the duration of each state (more on this point below). We will refer to the A-To-B and B-To-A memory molecules as *transition-encoding* memory molecules, because their abundance tracks switches, and the A-To-A and B-To-B memory molecules as *duration-encoding* memory molecules, because their abundance tracks the environmental state’s duration (a point we will return to below). We further assume that the memory molecules are degraded at a constant rate. Thus, the polymer assembly process will produce memory molecules that, if relatively stable, reflect the environment’s history of transitions, as we will see below. Note that the number of polymers that the system will construct will depend on the abundance of *T* monomers (which serve as the starting monomers for polymers). The number of polymers being assembled will in turn affect the abundance of memory molecules A-To-B and B-To-A, a point that we will return to below.

The additional operation needed for realizing inference is normalization-sampling (Figure 2B). Normalization-sampling means normalizing the transition counts, and then sampling from the resulting probability distribution. On a traditional digital computer, this is straightforward to implement sequentially. A standard algorithm for sampling from a probability vector θ (in effect, sampling from a multinomial distribution) is: calculate a cumulative sum up to each element $\theta_{i}$, then iterate through θ, draw a uniform random number w∈[0,1] for each entry $\theta_{i}$, and return the first $\theta_{i}$ such that $\sum_{j=1}^{i}\theta_{j}\geq w$. Within a cell, however, there is no readily accessible notion of sequential iteration to support this procedure; a concurrent mechanism is needed instead.

We can make use of the fact that concentrations lend themselves to encoding probabilities (also discussed in [22]) to derive a non-sequential version of the same computation. Consider two proteins, an activator and a repressor, that have expression levels $c_{1}$ and $c_{2}$, respectively, (Figure 2B). We can normalize and sample from the vector $\theta =[c_{1}\;c_{2}]$ by having the activator and repressor bind, in mutually exclusive fashion, a third molecule which we call an *integrator*. If the integrator is not in excess of the activator and repressor, then the fraction of activator-bound integrator will be proportional to the concentration of activator *normalized* by the sum of concentrations of activator and repressor (Figure 2B). This means that a molecular interaction that depends on the concentration of activator-bound integrator will occur in proportion to the probability $\frac{c_{1}}{c_{1}+c_{2}}$. This mechanism does not depend on sequential order: the interactions between activator, repressor, and integrator can all take place concurrently (similar integrator schemes have also used by other circuits that sense environmental changes in a way that is sensitive to order of exposure, e.g., [38]).

To encode the posterior probability of encountering *A* (or *B*) given the environment’s history, we can use the activator-repressor-integrator scheme to “normalize” the transition counts encoded by memory molecules (Figure 2C). When the environment is in state *A*, the A-To-A and A-To-B transition molecules will bind, in proportion to their abundance, to the A-To-B-integrator. The fraction of integrator molecules bound by A-To-B (relative to those bound by A-To-A) will represent $P(A|\begin{array}{c} \mathrm{history}) \end{array}$ (likewise for A-To-B and $P(B|\begin{array}{c} \mathrm{history}) \end{array}$. One complication is that the duration-encoding memory molecules, A-To-A and B-To-B, whose abundance is determined by the environmental state’s duration, will inevitably be more abundant than A-To-B and B-To-A. Thus, the rate constants governing the interactions between memory molecules and integrators have to be adjusted to account for this bias (see Appendix A).

We have implemented this circuit using Kappa, a rule-based language for describing and simulating stochastic biochemical systems (such simulations correspond formally to continuous-time Markov chains as described in [39,40]). Rule-based representations are especially suited for modeling polymerizing circuits such as this one, which may correspond to an intractably large number of ODEs (due to the combinatorial explosion of species  [41]). We next explore several features of this circuit when exposed to changing, probabilistic environments.

### 2.3. Circuit Behavior Reflects the Environment’s History

**Figure 3:**
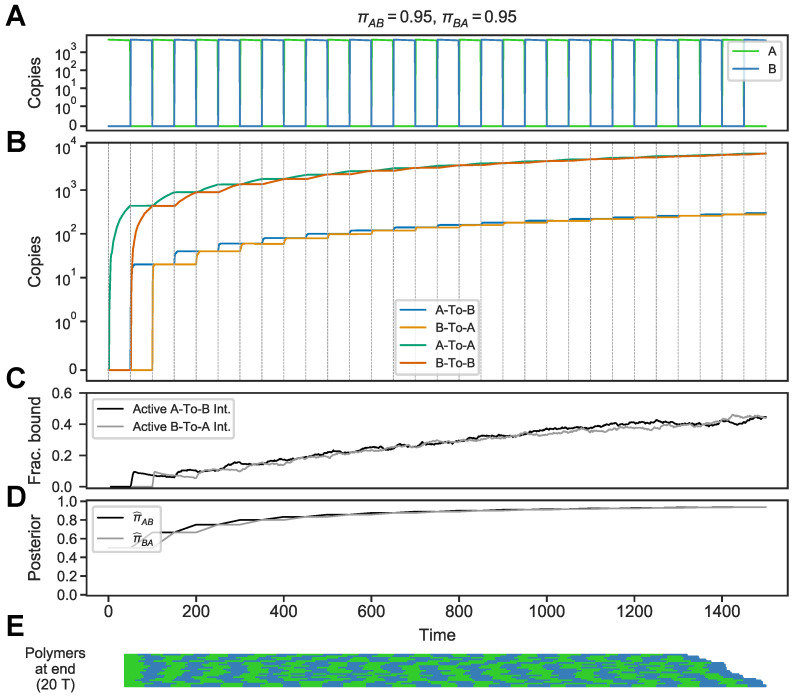
Stochastic simulation of the circuit in a periodic Markov chemical environment. (**A**) Environment in which *A* and *B* are pulsed and removed periodically, following a Markov model with transition probabilities $\pi_{AB}=\pi_{BA}=0.95$. (**B**) Levels of circuit components. Transition-encoding memory molecules A-To-B and B-To-A track transitions from *A* to *B* and *B* to *A*, respectively. Duration-encoding memory molecules A-To-A and B-To-B track the duration of states *A* and *B*, respectively. (**C**) Fraction of A-To-B integrator that is active, i.e., bound by A-To-B memory molecule (black), and fraction of A-To-B integrator that is active, i.e., bound by B-To-A memory molecule (grey). (**D**) Medians of posterior distribution over the transition probabilities $\pi_{AB}$ (black, ${\hat{\pi }}_{AB}$) and $\pi_{BA}$ (grey, ${\hat{\pi }}_{BA}$), calculated analytically using a discrete-time Markov model (each data point corresponding to a 50 time unit interval) with a uniform prior. (**E**) Polymers at the end of the simulation, plotted from head (left) to tail (right) and ordered by size from shortest (top) to longest (bottom). Stretches of green correspond to runs of *A*s, stretches of blue to runs of *B* (there is no relationship between this visualization of polymers and the time axis).

In order to simulate our model, we had to choose the number of polymers *T* that the circuit has to work with. The resulting circuit is sensitive to the environment’s history. When simulated (using Kappa) in a periodic environment ($\pi_{AB}=\pi_{BA}=0.95$), the circuit accumulates transition-encoding memory molecules at each switch (Figure 3A,B), as expected.

We next compared the circuit’s “posterior”—as encoded in the fraction of active integrators—to the posteriors based on the idealized, discrete-time Markov model. The posterior estimates of $\pi_{AB},\pi_{BA}$ in the idealized model are obtained by looking only at the sequence of switches, $\langle A,B,A,B,\ldots \rangle$, with a uniform prior over the two states (see Appendix A).

The fraction of active integrators qualitatively matches the posterior estimates of the idealized model (Figure 3C,D). As the circuit accumulates A-To-B memory molecules, for example, the fraction of active A-To-B integrators—i.e., those integrators bound by the A-To-B memory molecule—increases, following the analytic estimates of the transition probabilities (Figure 3C,D). Moreover, the linear polymers at the end of the simulation reflect the history of periodic switches (Figure 3E).

**Figure 4:**
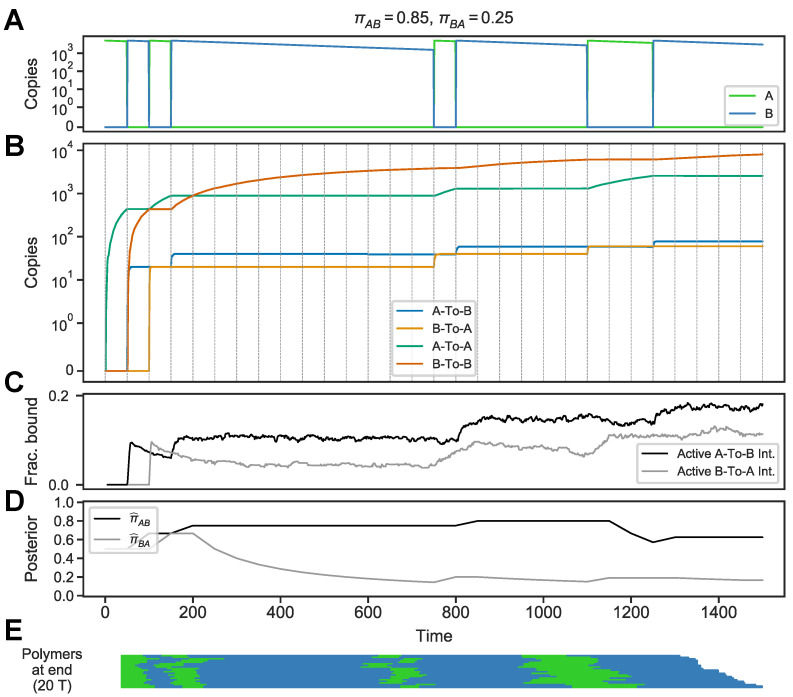
Circuit behavior in a “sticky” state environment. (**A**) Environment where transitions from *A* to *B* are frequent but the *B* state is “sticky” ($\pi_{AB}=0.85,\pi_{BA}=0.25$). (**B**) Levels of circuit components. (**C**) Fraction of active A-To-B and B-To-A integrators. (**D**) Posterior medians for transition probabilities $\pi_{AB}$ (black, ${\hat{\pi }}_{AB}$) and $\pi_{BA}$ (grey, ${\hat{\pi }}_{BA}$), calculated using a discrete-time Markov model with a uniform prior. (**E**) Polymers at the end of the simulation.

When exposed to an environment with different statistics, the circuit behaves accordingly. We considered an environment where *A* frequently switches to *B*, but switches from *B* to *A* are rare, meaning *B* is a “sticky” state ($\pi_{AB}=0.85,\pi_{BA}=0.25$) (Figure 4A). In this environment, the transition-encoding memory molecules track the switches, but the duration-encoding memory molecule B-To-B reflects that the environment tends to dwell in state *B* (Figure 4B). As a result, the fractions of active A-To-B and B-To-A integrators qualitatively track the analytic posteriors (Figure 4C,D). For instance, after the onset of *B* (time 50), the fraction of active B-To-A decreases as the environment stays in *B* (Figure 4C, time 50–750). The linear polymers assembled in this environment reflect this environment’s history (Figure 4E). Thus, the circuit is not limited to one particular Markov environment, but rather uses the process of polymer assembly to produce memory molecules that reflect the environment’s transition history.

### 2.4. Activity of Integrator Complexes Reflects Posterior Probabilities

**Figure 5:**
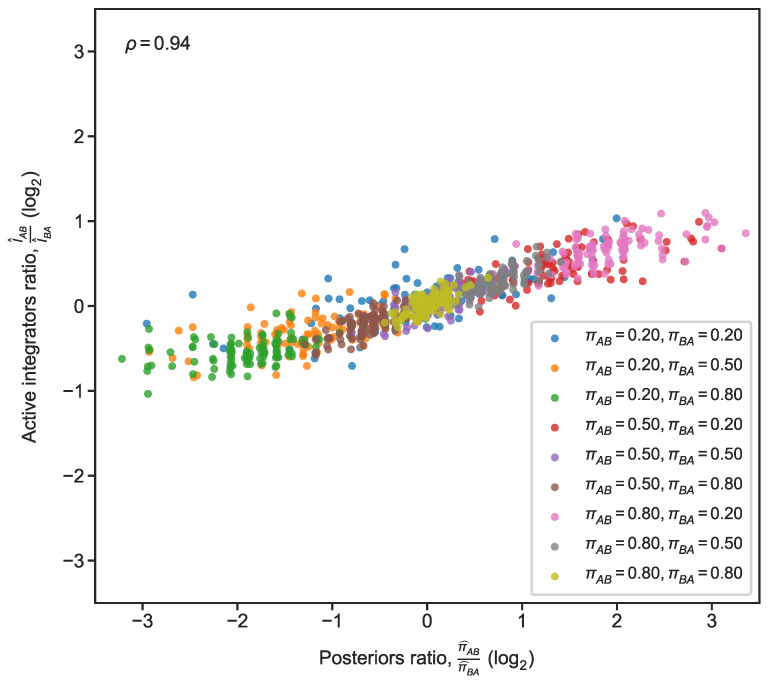
Comparison of circuit behavior with posterior estimates from idealized Markov model. Comparisons are shown for 9 different settings of $\pi_{AB},\pi_{BA}$ that were used to produce changing environments consisting of 50 perturbations (each with duration 50 time units). Each parameter assignment is plotted in a different color (100 simulations per assignment). On *x*-axis, ratio of posterior estimates ${\hat{\pi }}_{AB}$ and ${\hat{\pi }}_{BA}$ for $\pi_{AB}$ and $\pi_{BA}$, respectively. ${\hat{\pi }}_{AB}$ is the median of the posterior distribution over $\pi_{AB}$, and same for ${\hat{\pi }}_{BA}$ (*x*-axis values were jittered with random, small values for visualization purposes). On *y*-axis, ratio of the of active A-To-B integrator to that of active B-To-A integrator. ${\hat{I}}_{AB}$ is the fraction of active A-To-B integrators at the end of the environment (same for ${\hat{I}}_{BA}$).

We next compared our circuit’s behavior to the idealized probabilistic model more systematically, by exposing the circuit to many different assignments of the transition probabilities $\pi_{AB},\pi_{BA}$ (Figure 5). For each environment, we first estimated the median of the posterior over $\pi_{AB}$ at each time point and then took ${\hat{\pi }}_{AB}$ to be the median of those estimates (and similarly for $\pi_{BA}$ to obtain ${\hat{\pi }}_{BA}$). We then computed a similar quantity for our circuit by taking the median of the fraction of bound A-To-B integrators through time (${\hat{I}}_{AB}$) and likewise for B-To-A integrators to obtain ${\hat{I}}_{BA}$.

Since these quantities are not directly, quantitatively comparable, we compared the ratio of posteriors from the idealized model ($\frac{{\hat{\pi }}_{AB}}{{\hat{\pi }}_{BA}}$) with the ratio of the relevant active integrators ($\frac{{\hat{I}}_{AB}}{{\hat{I}}_{BA}}$). The comparison shows broad agreement (rank correlation ρ=0.94) across different assignments of transition probabilities (each assignment was simulated 100 times, Figure 5). It is important to note that this can only be a qualitative comparison, since the biological realization of the posterior and the analytic model begin from different starting points (zero active in the circuit’s case, versus a 50%-50% prior in the analytic model). Another caveat is these ratios being compared only reflect end-state results, not differences in the temporal trajectory of posterior ratios. We also stress again that the analytic model which operates in discrete-time is, by definition, an idealization that could never perfectly match the biological circuit which operates in continuous time and obviously lacks synchronous switches from one time point to another. In the next section, we describe some properties of our polymerizing circuit and its difference from the idealized discrete-time model.

### 2.5. Properties and Limitations of the Polymerizing Mechanism

While our circuit was guided by an analysis of inference in an idealized Markov model, these results indicate an important difference between that abstract model and our circuit as realized using polymerizing biochemical parts. Unlike in the abstract model we had started with, the circuit consumes the very “signal” that it is responding to, as *A*s and *B*s get incorporated into the circuit’s linear polymers. Thus, the circuit shapes the environment it inhabits, rather than passively observing its dynamics.

The magnitude of this effect will depend on the rate at which *A* and *B* are incorporated into polymers (*k* in Figure 2A) and the number of polymers being assembled, which is bounded by the abundance of *T* monomers. The rate of polymer assembly will in turn influence the rate of production of memory molecules. If *A* and *B* are abundant and *T* is small, as in Figure 3 and Figure 4 where there are 20 *T*s, then a single switch from, say, *A* to *B* can produce at most 20 A-To-B memory molecules. The concentration of *T*, therefore, influences the magnitude of the circuit’s memory (measured by copies of memory molecules), and this in turn affects the sensitivity and noise in the binding of integrators (in all of the above simulations, integrators are assumed to be fixed at 400 copies). The parameters governing polymer assembly, therefore, shape how responsive the circuit can be to environmental change.

The abundance of the circuit’s components relative to those of *A* and *B* also matters. In all of our simulations, *A* and *B* were far more abundant (5000 copies) than the number of polymers being assembled (10 *T*s), and we chose an appropriately small polymerization rate constant (k=0.0001; recall that *k* is the rate constant governing the addition of monomers to the end of a polymer). If *A* and *B* were in low abundance and/or the polymerization rate was really slow, then environmental switches would go undetected. On the other hand, if the polymerization rate was too high and/or the concentration of *T* too high, the circuit could in theory consume all the “nutrients” (using these to construct very long polymers), and therefore become insensitive to different durations (i.e., be unable to produce duration-encoding memory molecules). Naturally, the stability of the memory molecules produced through polymerization will determine how far back the circuit will “remember” environmental switches.

### 2.6. Environments with Changing Dynamics

While the periodic and “sticky” environments we have explored produce very different sequences of switches, both fall within our definition of Markov environments. Moreover, we have assumed that each environment follows the same set of transition probabilities throughout. We next asked how the circuit would behave in environment’s where there is a sudden change in the way that the environment changes (similar to the “meta-changing” environments described in [13]).

**Figure 6:**
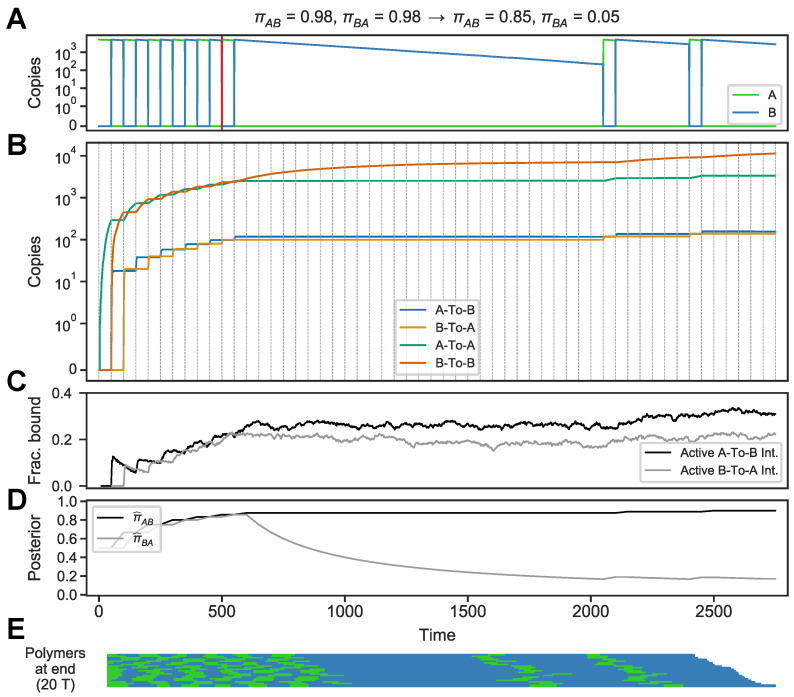
Circuit behavior in an environment that switches its dynamic. (**A**) Environment follows a periodic dynamic ($\pi_{AB}=\pi_{BA}=0.85$) and switches to a sticky *B* dynamic ($\pi_{AB}=0.85,\pi_{BA}=0.05$, marked by vertical red line). (**B**) Levels of circuit components. (**C**) Fraction of active A-To-B and B-To-A integrators. (**D**) Posterior medians for transition probabilities $\pi_{AB}$ (black, ${\hat{\pi }}_{AB}$) and $\pi_{BA}$ (grey, ${\hat{\pi }}_{BA}$), calculated using a discrete-time Markov model with a uniform prior. (**E**) Polymers at the end of the simulation.

In the environment shown in Figure 6, for example, the initial sequence of switches (time 0–500) is drawn from a periodic environment, $\pi_{AB}=\pi_{BA}=0.95$, and the remaining sequence from a “sticky” *B* environment, $\pi_{AB}=0.85,\pi_{BA}=0.05$ (Figure 6, time 500 through end). The circuit adapts to the new set of statistics, as seen in the changing curve of fraction of active integrators (Figure 6C, starting at time 500). The resulting polymers also correctly reflect the environment’s two distinct segments (Figure 6E). Naturally, the rate at which the circuit will adapt to such changes in the environment’s underlying dynamics will depend on how quickly the memory molecules are degraded.

### 2.7. Using Probabilistic Information to Regulate Other Processes

The probabilistic information obtained from the history of switches may be used to regulate other processes within the cell, such as a response to a specific environment. For this to be possible, as some cognitive scientists have argued, the information has to be “accessible” [42]. For accessibility, it is not sufficient to only have the system “contain” the information in its states in an information-theoretic sense (as was shown for memory in *B. subtilis* [16]). Rather, the information has to be instantiated in a form that is usable by other molecular processes within the cell.

**Figure 7:**
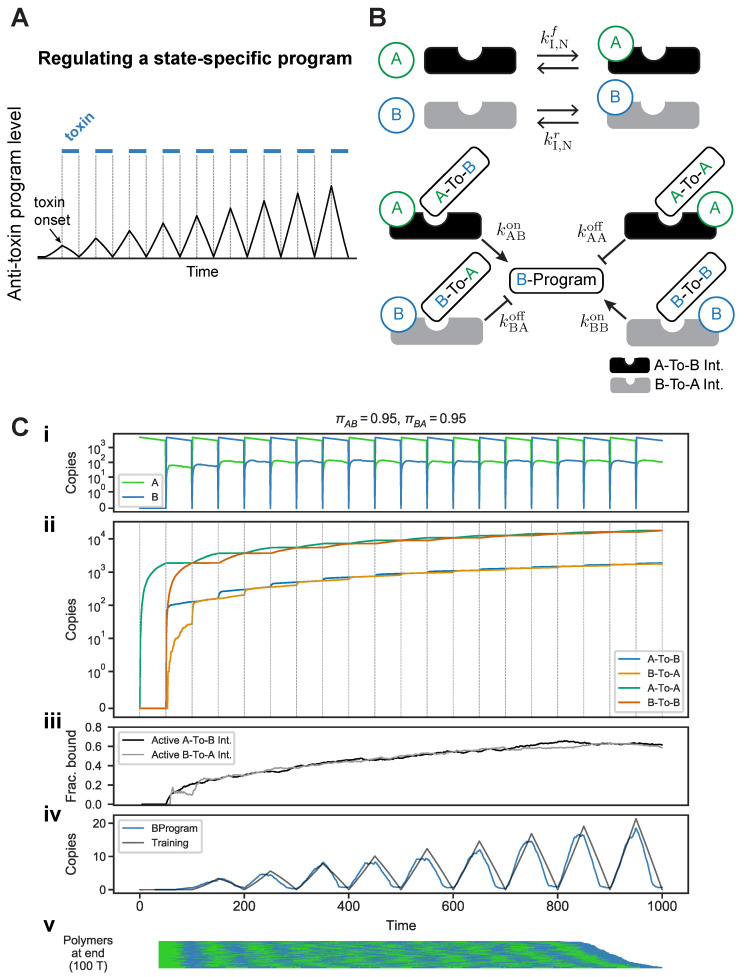
Regulating a state-specific program. (**A**) Hypothetical environment in which a toxin (blue horizontal lines) is pulsed periodically. An ideal expression profile for an anti-toxin program (black line): the program reaches peak expression at the onset of the toxin, and the maximal expression of the program increases as more switches between the toxin and non-toxin states are experienced. (**B**) A circuit for regulating a program specific to the *B* state, B-Program, and associated rate constants. (**C**) Behavior of circuit with state-specific program regulation, with rates optimized as defined in main text. (**i**) Levels of unbound *A* and *B*. Note that some *A* and *B* molecules are bound to integrators during a switch, which results in a mixture of *A*s and *B*s in the next pulse. (**ii**) Levels of circuit components. (**iii**) Fraction of active A-To-B and B-To-A integrators. (**iv**) The desired expression profile for B-Program (black line), used to optimize the rate constants, and the observed B-Program profile (blue line). (**v**) Transition history polymers at end of simulation (note the circuit was simulated with 100 *T* monomers).

We next show how the probabilistic information about the environment’s history can be used to regulate an environmental state-specific program, as shown in Figure 7A. In this scenario, the organism is probabilistically presented with a toxin, and (1) an anti-toxin program is required in order to increase chances of survival or growth, (2) the anti-toxin program takes time and substantial energy to produce (so it cannot be kept on at all times). Depending on the frequency of the toxin, the timescale of environmental change, and the associated metabolic costs, there are scenarios where it makes sense for organisms to anticipate the onset of the toxin and produce the program in advance ([13]; see also [43] for a seminal discussion of such trade-offs in changing environments).

We consider an abstract version of this problem in which a protein associated with the *B* state, B-Program (akin to the anti-toxin program), has to be produced prior to the onset of *B* in a periodic environment. We assume that the ideal expression profile of B-Program would one where B-Program is produced in advance, reaches its peak at the onset of *B*, and then degraded (as shown in Figure 7A). Moreover, as the circuit experiences more periodic switches, we expect the regulation of B-Program to become more pronounced, reflecting the fact that more “data” about the environment’s transition probabilities has been obtained.

To create this anticipatory behavior, we can utilize the posterior probability of encountering *B*, encoded in the integrator-memory molecule complexes. We designed a circuit that regulates B-Program in proportion to its posterior probability as encoded by these complexes (Figure 7B). This posterior probability is encoded in the fraction of A-To-B integrators that are active, i.e., bound by the A-To-B memory molecule. When *A* is present, it can bind these active integrators, forming a complex that produces B-Program (Figure 7B). However, when *A* binds an A-To-B integrator that is repressed (i.e., bound by A-To-A memory molecule), the resulting complex will degrade B-Program. When *B* is present, a symmetric circuit that uses the B-To-A integrator to enact the same logic with B-To-A and B-To-B memory molecules.

Crucially, the rate constants governing this circuit must match the timescales of change in the environment. If the desired behavior is to have the *A*-specific program (for example) reach its maximal expression prior to the onset of A, the circuit must be tuned to the average duration of *A* and *B* states. This “tuning” is reflected in the choice of rate constants. The rate constants for these interactions—which determine the binding of *A* and *B* to integrators, and the production/degradation of B-Program—have to be assigned in a way that produces the desired “sawtooth” expression profile shown in Figure 7A. We used an optimization procedure to set these rates (see Appendix A for details). With the resulting rate constants, the circuit indeed regulates B-Program in sawtooth-like fashion, where the regulation becomes more pronounced with increasing exposure to switches (Figure 7C). This behavior is consistent with the idea that more experiences of the environment lead to more confident estimates of transition probabilities (by accumulation of memory molecules), and that more confident estimates produce sharper responses to switches. Note that due to the regulation of B-Program, some of the *A* to *B* molecules remain bound to integrators during a switch, and are thus not removed, resulting in a mixture of *A* and *B* molecules in the next state. The number of molecules that can be carried over in this way is bounded by the number of integrator molecules.

## 3. Discussion

We have described a polymerizing circuit that performs probabilistic inference in a Markov model. Our analysis of the requirements of real-time inference in the Markov model led us to look for ways to biochemically realize two somewhat counter-intuitive operations: directional counting and normalization-sampling. The first was performed through polymer assembly, relying on the polarity of polymers and their constituents. The second was performed using a scheme where a pair of proteins bind to a third “integrator” protein in mutually exclusive fashion. With these two operations, the circuit can be used to regulate another biochemical process of interest, such as an environmental state-specific program.

The resulting circuit has several interesting properties. First, this circuit exhibits predictive behavior in a dynamic environment without relying on mutation and natural selection across generations. While mutation and selection can, from a mathematical perspective, implement solutions to probabilistic inference problems [44], and Darwinian evolution and inference algorithms have been argued to be conceptually similar strategies for solving the same basic problem [45], mutation and selection are nonetheless biologically distinct processes that operate on longer timescales than ontogenetic ones. The former operate on longer timescales; the latter can occur, theoretically, during the “lifetime” of a single cell experiencing environmental change. The ontogenetic behavior we explored is therefore distinct from laboratory evolution experiments with microbes or *in silico*-evolved circuits that were selected to fit a particular environment (although several previous studies have presented such experimental results as evidence of “predictive” cellular behavior [46,47], as argued in [30]).

Second, our circuit consumes the “signal” (*A* and *B*, in our case) that it anticipates. These molecules become part of the circuit (organism) through the process of polymer assembly. While we have referred to “the environment” as if separate from the circuit, this circuit actually alters the concentration of *A* and *B* (the magnitude of the effect depends on the relative abundances of nutrients and the rate of polymer assembly). This demonstrates the blurry line between metabolism and signal transduction [48].

Since the circuit records the history of change in polymers, this opens up possibilities for molecular mechanisms that utilize these sequences by interacting with the polymers. A variety of molecular mechanisms, such as kinesin motors that walk along microtubules—which have been intensively studied experimentally [49] and theoretically [31]—can be a source of inspiration. Microtubules can also be regulated through myriad post-translational modifications to tubulin proteins, which have been viewed as a kind of combinatorial “tubulin code” [50,51,52]. These modifications can affect the localization and size of microtubules by tuning the binding of microtubule-regulating proteins. Such mechanisms can also be explored as forms of biological information-processing.

A theoretical study of these mechanisms would benefit from further development of formal languages for representing and simulating combinatorial biochemistry, such as Kappa [40]. Kappa, for example, currently does not explicitly model physical space, which raises a challenge: how to capture more of the ways in which physical space constrains and shapes polymerization—in a dividing cell, for instance—while keeping the formal language sufficiently abstract so as to be interpretable, and the simulations tractable? Computational tools for visualizing polymer assembly, through time and space, could also help the study of polymerization as a form of information-processing.

Several open questions about polymerization and inference also remain. It would be interesting to explore how the circuit design we have proposed can be adapted to more complex environments. A recent study has investigated how inference in hidden Markov Models can be performed using CRNs [53], and it would be worth exploring whether a polymerization scheme for real-time inference of the sort we have developed could be used for these more complex Markov models that have latent states. Similarly, it may be fruitful to explore how circuits could cope with “meta-changing” environments whose dynamics change according to yet another unobserved stochastic process [13]. Another avenue would be to explore the energetic costs of such inference-performing circuits, and how they might be integrated into a larger cellular model that accounts for cell division, growth, and the cell’s self-producing (“autopoietic”) capacity [54,55].

## Figures and Tables

**Figure A1:**
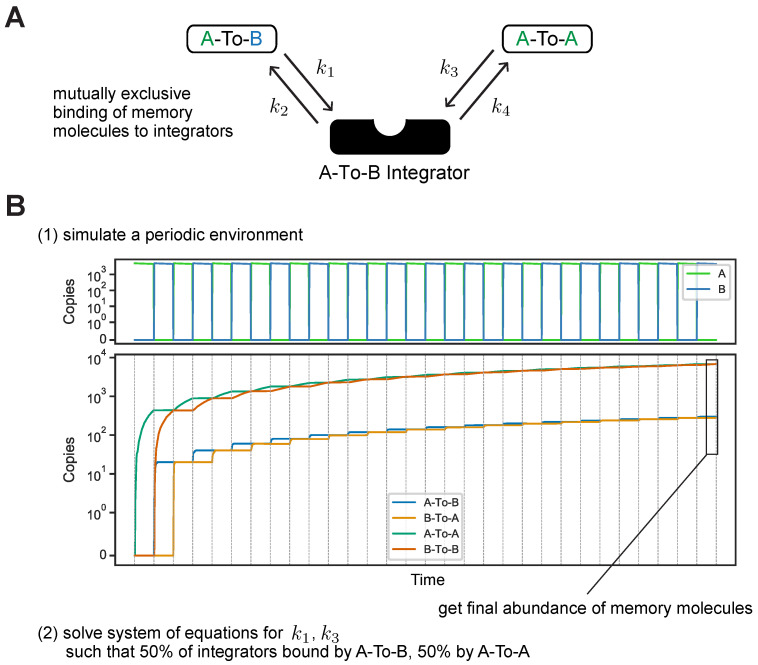
Adjustment of rate constants for the normalization-sampling circuit. (**A**) Rate constants to be adjusted for the A-To-B integrator. An equivalent set of rates for B-To-A integrator is not shown. (**B**) Procedure for adjusting the rate constants by first simulating a specific periodic environment to obtain the level of memory molecules, and then analytically solving for the desired rate constants as described in Appendix A.

## Appendix A. Methods

### Appendix A.1. Real-Time Inference in a Discrete-Time Markov Model

We briefly describe an algorithm for real-time inference in a Markov model (which was used to generate the analytic estimate of the posterior in all figures). We assume a first-order, discrete-time Markov model over two discrete states, *A* and *B*, which is characterized by transition probabilities $\pi_{AB}$ and $\pi_{BA}$ (where $\pi_{AA}=1-\pi_{AB}$ and $\pi_{BB}=1-\pi_{BA}$). A sequence of observations is generated from the model by first sampling an initial state $X_{0}$ (from a uniform prior over *A* and *B*) and sampling subsequent observations $X_{1},X_{2},X_{3},\ldots$ using the transition probabilities:

- If $X_{t}=A$, sample $X_{t+1}∼\mathrm{Bern}\left(1-\pi_{AB}\right)$
- Otherwise, if $X_{t}=B$, sample $X_{t+1}∼\mathrm{Bern}\left(\pi_{BA}\right)$.

The quantity of interest here is the posterior distribution over transition probabilities:

$$\begin{array}{cc} P(\pi_{AB},\pi_{BA}|\begin{array}{c} \mathrm{history}) \end{array} & \propto \mathrm{likelihood}\times \mathrm{prior}\\ \; & \propto P\left(\begin{array}{c} \mathrm{history}| \end{array}\pi_{AB},\pi_{BA}\right)P\left(\pi_{AB},\pi_{BA}\right) \end{array}$$

where history stands for the sequence of observations of past states, $\langle X_{t},X_{t-1},\ldots \rangle$. A standard approach is to use independent priors over the transition probabilities, which lets us treat the posterior for each parameter separately:

$$\begin{array}{cc} P(\pi_{AB}|\begin{array}{c} \mathrm{history}) \end{array} & \propto P\left(\begin{array}{c} \mathrm{history}| \end{array}\pi_{AB}\right)P\left(\pi_{AB}\right)\\ P(\pi_{BA}|\begin{array}{c} \mathrm{history}) \end{array} & \propto P\left(\begin{array}{c} \mathrm{history}| \end{array}\pi_{BA}\right)P\left(\pi_{BA}\right) \end{array}$$

A convenient choice of prior over transition probabilities is the Beta distribution. A Beta distribution, $\mathrm{Beta}(\theta ;\alpha_{0},\alpha_{1})$, over θ∈[0,1] is determined by a pair of shape parameters $\alpha_{0},\alpha_{1}$. Different settings of the alphas can be used to encode distinct beliefs about θ. As examples: $\alpha_{0}=\alpha_{1}=1$ is equivalent to a uniform distribution over [0,1], $\alpha_{0}=\alpha_{1}=100$ is a unimodal distribution whose mode is at θ=0.5, and $\alpha_{0}<1,\alpha_{1}<1$ (e.g., $\alpha_{0}=\alpha_{1}=0.5$) is a U-shaped distribution corresponding to the belief that θ is likely to be close to 0 or 1. The Beta distribution is conjugate to the binomial distribution [56], meaning that a posterior that is the product of a Beta prior and a binomial likelihood equals another Beta distribution whose parameters can be calculated exactly.

The observations of the environment, history, can be compressed into a set of counts (as described in main text) that are binomially distributed, which allows us to use the Beta-Binomial conjugacy to calculate our posteriors of interest analytically. For a two-state Markov model, the relevant counts are: (1) *c_AB_*, the number of *A*→*B* transitions, (2) *c_BA_*, the number of *B*→*A* transitions, and (3) the number of total switches *s* in history. (Note that the other entries of the transition counts matrix T can be computed from this pair of summary statistics and *s*: $c_{AA}=s-c_{AB}$ and $c_{BB}=s-c_{BA}$.) We can then expand the posterior over $\pi_{AB}$ as follows:

$$\begin{array}{cc} P(\pi_{AB}|\begin{array}{c} \mathrm{history}) \end{array} & \propto P\left(\begin{array}{c} \mathrm{history}| \end{array}\pi_{AB}\right)P\left(\pi_{AB}\right)\\ \; & \propto P\left(c_{AB},s|\pi_{AB}\right)P\left(\pi_{AB}\right)\\ \; & \propto \mathrm{Bin}\left(C_{AB};\pi_{AB},s\right)\mathrm{Beta}\left(\pi_{AB};\alpha_{0},\alpha_{1}\right)\\ \; & =\mathrm{Beta}(\pi_{AB};C_{AB}+\alpha_{0},s-C_{AB}+\alpha_{1}) \end{array}$$

(and similarly for $\pi_{BA}$.)

This posterior can be computed in real-time up through the *k*th observation (a task which is called “filtering” [57]) using a recursive procedure that proceeds as follows. Let $C_{AB}^{k}$ be the number of A→B transitions up to the *k*th observation, and let $b_{\pi_{AB}}^{k}$ be the posterior up to the *k*th observation. The base case of the procedure is the first observation, which is the switch from $X_{0}\to X_{1}$ (where k=1):

$$\begin{array}{cc} b_{\pi_{AB}}^{1} & =P(\pi_{AB}|\langle X_{1},X_{0}\rangle )\\ \; & \propto \mathrm{Bin}\left(C_{AB}^{k};\pi_{AB},k\right)\mathrm{Beta}\left(\pi_{AB};\alpha_{0},\alpha_{1}\right)\\ \; & =\mathrm{Beta}(\pi_{AB};C_{AB}^{k}+\alpha_{0},s-C_{AB}+\alpha_{1}) \end{array}$$

We can now define the $b_{\pi_{AB}}^{k+1}$ in terms of $b_{\pi_{AB}}^{k}$ by noting that the posterior as of the *k*th observation, $b_{\pi_{AB}}^{k}$, serves as the prior at k+1. Let $\alpha_{0}^{k},\alpha_{1}^{k}$ be the parameters of $b_{\pi_{AB}}^{k}$. Now let $I_{AB}$ be an indicator variable that is 1 if $X_{k}\to X_{k+1}$ is an A→B transition (and 0 otherwise), and $I_{AA}$ an indicator variable that is 1 if $X_{k}\to X_{k+1}$ is an A→A transition (and 0 otherwise). Then the posterior after the k+1 observation is:

$$\begin{array}{c} b_{\pi_{AB}}^{k+1}=\mathrm{Beta}\left(\pi_{AB};\alpha_{0}^{k}+I_{AB},\alpha_{1}^{k}+I_{AA}\right) \end{array}$$

Note that if $X_{k}=B$, then $I_{AB}=I_{AA}=0$, which means the estimate of the posterior over $\pi_{AB}$ does not change (i.e., $b_{AB}^{k+1}=b_{AB}^{k}$). The same derivation as above applies to the posterior over $\pi_{BA}$.

To estimate the posterior over the transition probabilities in the above figures (e.g., Figure 3D), we used the median of the posterior distribution at each time step.

### Appendix A.2. Adjustment of Rate Constants for Normalization-Sampling Circuit

As noted in the main text, the rate constants for the normalization-sampling circuit, which control the binding of integrators to memory molecules, have to be adjusted to account for the fact that memory molecules encoding transitions ( A-To-B and B-To-A ) will inevitably be less abundant than the memory molecules encoding durations ( A-To-B and B-To-A ). The four rate constants to be adjusted for the A-To-B integrator are shown in Figure A1 (the analogous set of rate constants for B-To-A integrator are not shown). The normalization-sampling circuit for the A-To-B integrator is modeled by the following reactions:

$$\begin{array}{cc} M_{AB}+I & \overset{k_{1}}{\underset{k_{2}}{⇌}}C\\ M_{AA}+I & \overset{k_{3}}{\underset{k_{4}}{⇌}}D \end{array}$$

where $M_{AB}$ and $M_{AA}$ are the species of unbound A-To-B and A-To-A memory molecules (respectively), *I* the unbound integrator, *C* the integrator-bound A-To-B memory molecule, and *D* the integrator-bound A-To-A memory molecule. In order to accurately reflect the environment’s statistics, the transition-encoding memory molecules for the integrators should be higher than those of duration-encoding memory molecules. But how much higher?

We selected rate constants that, in a specific environment that switches evenly between *A* and *B* for *N* pulses, would result in half of the A-To-B integrator molecules bound by A-To-B and the other half by A-To-A (and likewise for B-To-A integrators). Such an assignment of rate constants means that the circuit accurately reflects the environment’s statistics of equal switching, and that after *N* pulses, half of the integrators are occupied. (The latter is a reasonable but somewhat arbitrary choice; depending on the abundance of integrators and the nutrients in the environment, one could choose rate constants that result in a different fraction of the integrators being used.)

To assign the rate constants, we used the following combination of simulation and analytic calculation. We first simulated our circuit, initialized with 20 T monomers and 400 integrators, on a perfectly periodic environment ($\pi_{AB}=1.0,\pi_{BA}=1.0$) consisting of 30 pulses (each with duration of 50 time units). We then obtained the resulting abundance of memory molecules (A-To-A, A-To-B, B-To-A, B-To-B).

We then expressed the normalization-sampling circuit described above as the following system of equations, which we represented in the computer algebra system Sympy [58]:

$$\begin{array}{cc} {M_{AB}}_{0}-C-M_{AB} & =0\\ {M_{AA}}_{0}-D-M_{AA} & =0\\ I_{0}-C-D-I & =0\\ k_{1}M_{AB}I-k_{2}C & =0\\ k_{3}M_{AA}I-k_{4}D & =0\\ \frac{C}{M_{AB}I}-\frac{k_{1}}{k_{2}} & =0\\ \frac{D}{M_{AA}I}-\frac{k_{3}}{k_{4}} & =0\\ \frac{C}{C+D}-0.5 & =0 \end{array}$$

where ${M_{AB}}_{0},{M_{AA}}_{0},I_{0}$ are the initial amounts of (unbound) memory molecules and integrators, respectively. We plugged in $I_{0}=400$, and the abundances of the memory molecules from our simulation on a periodic environment for ${M_{AB}}_{0},M{{}_{AA}}_{0}$. Since we aim to have half of the integrators bound by $M_{AB}$ and the other half by $M_{AA}$, we set $C=D=\frac{1}{2}I_{0}$. We then assumed that the backward rate constants are equal and set them to a reasonable value ($k_{2}=k_{4}=0.005$) and solved for $k_{1},k_{3}$ using Sympy. For the specific periodic environment we used, this calculation produces $k_{1}/k_{3}\approx 45$.

### Appendix A.3. Stochastic Simulations

All stochastic simulations of the circuit were performed using the Kappa simulator, KaSim (version: v4.1-8-ge04a210d8). Example Kappa programs used in the analyses:

- Kappa program for circuit in periodic environment (https://gist.github.com/yarden/3fda6f13d1af7d7b628c0a4b48a04529 (accessed on 12 July 2021.))
- Kappa program for circuit with regulation of B-Program (https://gist.github.com/yarden/8de42a93381d6c364bbc93293a56b4e4 (accessed on 12 July 2021.))

### Appendix A.4. Optimizing Rate Constants for State-Specific Program Regulation

To regulate B-Program in a sawtooth-like manner (Figure 7A), we searched for an assignment of the six parameters shown in Figure 7B: rate constants for integrator binding to *A* or *B* ($k_{I,N}^{f},k_{I,N}^{r}$), and for the production/degradation of B-Program by complexes of integrators, memory molecules, and *A* or *B* ($k_{AB}^{on},k_{AA}^{off},k_{BA}^{off},k_{BB}^{on}$). While it would be more computationally efficient to search over this six-dimensional space by converting our Kappa program to ODEs, our circuit polymerizes and so cannot be accurately simulated as ODEs. We therefore used KaSim to stochastically simulate the full Kappa program for our circuit. We therefore optimized the rate constants vector $R=[k_{I,N}^{f},k_{I,N}^{r},k_{AB}^{on},k_{AA}^{off},k_{BA}^{off},k_{BB}^{on}]$ by working with the circuit as represented in Kappa, simulated using KaSim.

We defined a sum of squares loss function, L(y,s), to score a simulated values of the B-Program, $y=\langle y_{0},\ldots ,y_{T}\rangle$, against the desired sawtooth-signal $s=\langle s_{0},\ldots ,s_{T}\rangle$, where t∈{0,…,T} are the simulation time bins: $L\left(y,s\right)=\sum_{t=0}^{T}\left|y_{t}-s_{t}\right|$. To compute the loss for a given assignment of *R*, we simulated the circuit with these assignments and scored the resulting values of B-Program (smoothed with 3 time unit sliding window) against the sawtooth signal using *L*. We optimized *R* using scipy’s minimize function [59] (initialized with a reasonable guess).
